# Gene expression signature for predicting homologous recombination deficiency in triple-negative breast cancer

**DOI:** 10.1038/s41523-024-00671-1

**Published:** 2024-07-19

**Authors:** Jia-Wern Pan, Zi-Ching Tan, Pei-Sze Ng, Muhammad Mamduh Ahmad Zabidi, Putri Nur Fatin, Jie-Ying Teo, Siti Norhidayu Hasan, Tania Islam, Li-Ying Teoh, Suniza Jamaris, Mee-Hoong See, Cheng-Har Yip, Pathmanathan Rajadurai, Lai-Meng Looi, Nur Aishah Mohd Taib, Oscar M. Rueda, Carlos Caldas, Suet-Feung Chin, Joanna Lim, Soo-Hwang Teo

**Affiliations:** 1 Cancer Research Malaysia, Subang Jaya, Malaysia; 2 Roche Services (Asia Pacific), The Pinnacle, Bandar Sunway, Subang Jaya, Malaysia; 3 Department of Surgery, Faculty of Medicine, University Malaya, Kuala Lumpur, Malaysia; 4 Subang Jaya Medical Centre, Subang Jaya, Malaysia; 5 Jeffrey Cheah School of Medicine & Health Sciences, Monash University Malaysia, Jalan Lagoon Selatan, Bandar Sunway, Subang Jaya, Malaysia; 6 Department of Pathology, Faculty of Medicine, University Malaya, Kuala Lumpur, Malaysia; 7 Cancer Research UK, Cambridge Institute & Department of Oncology, Li Ka Shing Centre, Robinson Way, Cambridge, UK; 8 NIHR Cambridge Biomedical Research Centre and Cambridge Experimental Cancer Medicine Centre, Cambridge University Hospital NHS Foundation Trust, Cambridge, UK; 9 University Malaya Cancer Research Institute, Faculty of Medicine, University Malaya, Kuala Lumpur, Malaysia

**Keywords:** Breast cancer, Cancer genomics, Cancer genetics, Predictive markers, Tumour biomarkers

## Abstract

Triple-negative breast cancers (TNBCs) are a subset of breast cancers that have remained difficult to treat. A proportion of TNBCs arising in non-carriers of *BRCA* pathogenic variants have genomic features that are similar to *BRCA* carriers and may also benefit from PARP inhibitor treatment. Using genomic data from 129 TNBC samples from the Malaysian Breast Cancer (MyBrCa) cohort, we developed a gene expression-based machine learning classifier for homologous recombination deficiency (HRD) in TNBCs. The classifier identified samples with HRD mutational signature at an AUROC of 0.93 in MyBrCa validation datasets and 0.84 in TCGA TNBCs. Additionally, the classifier strongly segregated HRD-associated genomic features in TNBCs from TCGA, METABRIC, and ICGC. Thus, our gene expression classifier may identify triple-negative breast cancer patients with homologous recombination deficiency, suggesting an alternative method to identify individuals who may benefit from treatment with PARP inhibitors or platinum chemotherapy.

## Introduction

Triple negative breast cancer (TNBC) continues to be an area of unmet clinical need, as this aggressive subtype has fewer treatment options and higher mortality rates compared to other subtypes of breast cancer^1–3^. As a “catch-all” diagnosis for all breast tumours that test negative for both hormone receptors and HER2, TNBC is highly heterogeneous, and can be divided into several subtypes of its own^4,5^, each with potentially different treatment options. In this context, one potential biomarker that may be useful for subcategorizing TNBC patients for treatment is homologous recombination deficiency (HRD).

Cells with homologous recombination deficiency (HRD) have defects in their homologous recombination pathway leading to a diminished ability to repair DNA damage. In breast cancer, HRD is an important biomarker for therapies that utilize DNA-damaging agents such as platinum chemotherapy and PARP inhibitors. Platinum chemotherapies such as cisplatin, oxaliplatin, and carboplatin induce crosslinking of DNA that inhibits DNA repair and synthesis^6^. PARP inhibitors, on the other hand, are a new class of drugs that inhibit the action of Poly (ADP-ribose) polymerase (PARP) proteins, leading to double strand DNA breaks during cellular replication^7^. In tumours with HRD, these DNA-damaging agents cause an accumulation of mutations, leading to synthetic lethality and eventually cell death^8^. Tumours with deleterious genomic *BRCA* variants have defective homologous recombination repair pathways^9^, and PARP inhibitors have been approved for TNBC for individuals with deleterious germline variants in *BRCA1/2* (g*BRCA*m)^10,11^.

Besides deleterious germline *BRCA* variants, other molecular alterations in tumours may also lead to similar defects in the HRD pathway. This “BRCAness” feature has been proposed to broaden the patient population to PARP inhibitors^12^, and various molecular aspects of “BRCAness” have been characterized^13^. Some breast tumours with the “BRCAness” feature may arise when expressions of *BRCA1* or *BRCA2* are repressed by hypermethylation or somatic mutation^14^, or when the homologous recombination pathway is abrogated through mutations in other genes in the pathway (e.g. *PALB2*)^15,16^, and there are ongoing clinical studies that seek to expand the utility of PARP inhibitors in this context^17^. In addition, transcriptional signatures and genomic mutational signatures have been generated to identify tumours with HRD, with some association with PARP inhibitor sensitivity^18,19^. Clinical studies to examine the utility of these genomic signatures as predictive biomarkers have also been initiated^20^. In other cancer settings such as recurrent or high grade serous ovarian carcinoma, HRD assays such as the myChoice HRD assay and the Foundation Medicine T5 NGS assay have demonstrated some utility in guiding treatment with PARP inhibitors, and have received FDA approval as companion diagnostics^21–23^.

We have recently characterized the genomic and transcriptomic profiles of fresh frozen breast tumours from a large cohort of Malaysian patients of Chinese, Malay and Indian descent (the MyBrCa cohort)^24^. In order to study transcriptomic biomarkers for HRD in Asian TNBC, we first defined HRD status by clustering our TNBC samples using genomic features associated with HRD, followed by differential gene expression analyses comparing samples with high HRD to samples with low HRD. We identified a set of largely novel genes that were associated with HRD in our cohort, which we used to train a machine learning classifier to classify patient tumour samples as having high or low HRD. We validated the classifier using TNBC samples from the TCGA, ICGC, and METABRIC cohorts. We also validated the classifier on an alternative NanoString platform, using both FFPE and fresh frozen tumour samples. This classifier may have clinical utility as a non-gBRCAm biomarker to select for patients with high HRD who may benefit from treatment with platinum chemotherapy or PARP inhibitors.

## Results

### Study population

Our discovery and training dataset consisted of 129 TNBC samples from the MyBrCa cohort, for which whole-exome sequencing and RNA-seq data were derived from fresh frozen primary tumour tissue and matched blood samples. 94 of these samples have been published previously (Pan et al.^24^), while the remaining 35 were sequenced more recently and have not been formally described. Of the 94 previously published samples, eight samples had pathogenic germline *BRCA1* variants, two samples had pathogenic germline *BRCA2* variants, and two samples had pathogenic germline *PALB2* variants^25^. The average age of the 129 TNBC patients was 52.9 years ( ±13.6), and the majority of the samples were Stage II or Stage III, poorly-differentiated invasive breast carcinomas of no special type (NST) (Table 1). Our validation cohorts comprised of 87 TNBC tumours from The Cancer Genome Atlas (TCGA) breast cancer cohort^26^ (TCGA 2012), 306 TNBC tumour samples from the Molecular Taxonomy of Breast Cancer International Consortium (METABRIC) breast cancer cohort^27,28^, and 73 TNBC tumour samples from the Nik-Zainal (2016) breast cancer cohort (NZ-560)^29^.

**Table 1:** Clinico-pathological characteristics of the MyBrCa TNBC cohort

	MyBrCa TNBC Overall	MyBrCa TNBC HRD High	MyBrCa TNBC HRD Low	Statistical significance
Subjects (*n*)	129	41	72	
Patient age (yr ± SD)	52.9 ± 13.6	48.1 ± 11.7	56.5 ± 14.3	*p* = 0.001
TNM stage (*n*(%))				*p* = 0.83
I	20 (15.5)	7 (17.1)	11 (15.3)	*p* = 0.78
II	62 (48.1)	22 (53.7)	33 (45.8)	*p* = 0.39
III	41 (31.8)	11 (26.8)	25 (34.7)	*p* = 0.40
IV	2 (1.6)	0 (0.0)	2 (2.8)	*p* = 0.91
N/A	4 (3.1)	1 (2.4)	1 (1.4)	
Tumour Grade (*n*(%))				*p* = 0.31
1	0 (0.0)	0 (0.0)	0 (0.0)	
2	20 (15.5)	5 (12.2)	13 (18.1)	*p* = 0.31
3	91 (70.5)	33 (80.5)	48 (66.7)	*p* = 0.31
N/A	18 (14.0)	3 (7.3)	11 (15.3)	
Histology subtype (*n*(%))				*p* = 0.93
Lobular Carcinoma	2 (1.6)	0 (0.0)	2 (2.8)	*p* = 0.91
Carcinoma of no specific type (NST)	106 (82.1)	34 (82.9)	60 (83.3)	*p* = 0.87
Other	7 (5.4)	3 (7.3)	4 (5.6)	*p* = 0.69
N/A	14 (10.9)	4 (9.8)	6 (8.3)	

Statistical significance was calculated using student’s t-test for patient age and chi-square tests for all other variables, with N/A values excluded. 16 TNBC samples did not have a consensus HRD designation and were excluded from the subgroup analyses.

### HRD in Asian TNBC

First, we examined the prevalence of HRD in the Asian TNBC setting by conducting an unsupervised clustering analysis of several genomic features associated with HRD in the MyBrCa TNBC samples. These features include commonly accepted features of HRD such as genomic loss of heterozygosity (LOH), telomeric allelic imbalance (TAI) and large-scale state transition (LST), short indels, copy number aberrations (CNAs) including gene amplification, gain, loss, and deletion, and the COSMIC mutational signature SBS3.

**Fig. 1:**
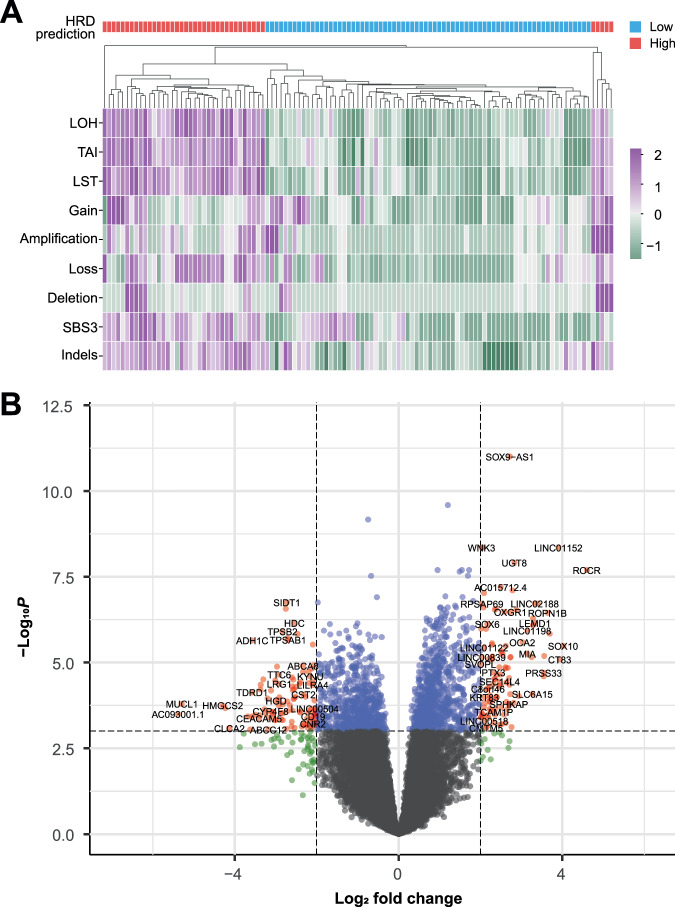
Clustering and gene expression analyses for homologous recombination deficiency (HRD) in MyBrCa TNBC samples. **A** Unsupervised hierarchical clustering of 129 MyBrCa TNBC samples using HRD-associated features including the COSMIC single base substitution mutational signature 3 (SBS3), short insertions and deletions (indels), loss-of-heterozygosity (LOH), telomeric allelic imbalance (TAI), large-scale transitions (LST), as well as copy number amplifications, deletions, gain and loss. All scores were scaled using z-scores, and the indels score was also log-transformed prior to scaling. Designations for each sample as HRD High or HRD Low are indicated by the “HRD prediction” annotation bar. **B** Volcano plot for differential expression analysis comparing HRD High and HRD Low samples. Dotted lines indicate the thresholds used to classify genes as differentially expressed (Benjamini-Hochberg adjusted *p*-value < 0.001, absolute log_2_ fold change > 2).

Hierarchical (Fig. 1A) and k-means (Supp. Fig. 2) clustering of these genomic features revealed two distinct clusters which we called HRD High and HRD Low based on the levels of these HRD-associated scores. Out of the 129 samples that were clustered, 113 samples were concordant between the two clustering algorithms, while 16 samples were discordant and dropped from subsequent analyses. Of the 113 concordant samples, 41 (32%) were categorized as HRD High and 72 (68%) were categorized as HRD low.

Next, using differential gene expression analysis of RNA-sequencing data from the TNBC tumour samples, we identified a set of 217 genes that were differentially expressed between the two groups by manually curating the top upregulated and downregulated genes based on Benjamini-Hochberg adjusted *p*-values of less than 0.001, with a minimum log_2_ fold change of 2 in either direction (Fig. 1B, Supp. Table 2). This gene set of 217 genes was substantially different from previous HRD-associated gene sets derived in studies by Peng et al.^30^ and the i-SPY 2 consortium^31,32^, with very small numbers of overlapping genes (1 between MyBrCa and Peng, 15 between MyBrCa and i-SPY 2).

Over-representation analysis of the selected 217 differentially expressed genes using KEGG and Reactome pathway-based gene sets revealed an enrichment in a number of metabolic and signaling pathways (Supp. Table 3), but not pathways known to be associated with HRD. Similarly, analysis of over-represented gene ontology (GO) terms for the 217 genes showed an enrichment of extracellular matrix, plasma membrane, and hormone-related terms, but not HRD-related terms (Supp. Table 4), suggesting that the 217 genes were largely not previously known to be associated with HRD. On the other hand, gene set enrichment analysis (GSEA) of MSigDB Hallmark and KEGG pathways comparing TNBC tumour samples classified as HRD High to samples classified as HRD Low revealed an upregulation of cell cycle and DNA repair pathways in samples classified as HRD High, similar to previous studies^13^, suggesting that HRD pathways are indeed differentially expressed between the two groups when looking across the whole transcriptome (Supp. Tables 5-6). Interestingly, immune-related pathways appear to be downregulated in the HRD High group and upregulated in the HRD Low group as well (Supp. Tables 5-6).

### Classification of tumour samples according to HRD status using the gene set

**Fig. 2:**
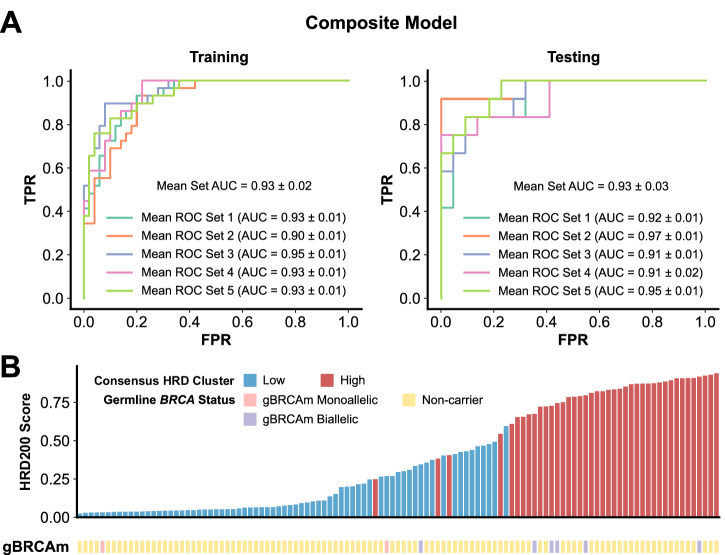
Performance of the HRD200 classifier in the MyBrCa TNBC cohort. **A** Receiver operating characteristic (ROC) curves of false positive rate (FPR) and true positive rate (TPR) showing the performance of the HRD200 composite classifier in predicting HRD High status in 70:30 training:testing gene expression datasets from 113 MyBrCa TNBC samples. The HRD200 classifier was trained on gene expression data of 217 differentially expressed genes. The ROC curves for each of the five shuffled 70:30 datasets are shown separately. **B** Bar chart showing the probability of a sample being HRD High according to the HRD200 classifier, compared to their HRD classification by consensus clustering (color of the bar) and known germline *BRCA* status (“gBRCAm” annotation).

Using the set of 217 genes identified above, we trained a machine learning classifier, which we call HRD200, to classify the HRD status of any given tumour sample, using an adaptation of the composite classifier framework described in Sammut et al.^33^. Our adaptation of this framework incorporates 5-fold stratified shuffling to split the samples along 70/30 ratio for model training and testing, respectively, followed by feature selection and 5-fold cross-validation (see Methods, Supp. Table 7). The composite classifier under this framework achieved a mean AUROC of 0.93 in both training and testing datasets, utilizing 122 of the 217 genes as features (Fig. 2A). Using a probability cut-off that maximized F1 score (0.5), the HRD200 ensemble classifier designated 38 out of the 41 Asian TNBC in the MyBrCa samples with high HRD scores correctly as “HRD High” and classified 71 out of 72 of the samples with low HRD scores correctly as “HRD Low”, for an F1 score of 0.97 and an accuracy of 96%. The classifier also categorized 5 out of the 6 samples in our cohort with known biallelic pathogenic germline BRCA1 variants as HRD High (Fig. 2B). In addition, the use of our specific 217 gene set with this classification approach was able to outperform similar classifiers using the gene sets by Peng and i-SPY 2, although the difference was small (Supp. Fig. 3).

Given that the classifier was trained with the 217 genes that were identified using the entire MyBrCa data set, there was likely some information leakage that may have led to overly optimistic results with the testing datasets. Thus, to validate our methodology, we also trained the classifier using other sets of 200 genes, using a sliding window approach to derive gene sets of increasing informativeness based on their differential expression. We found that more significantly differentially expressed genes produced classifiers with high AUROCs and vice versa (Supp. Fig. 4), showing that the informativeness of the gene set used for the model directly influences the classifier’s predictive ability. Interestingly, we found that we could also use random sets of 200 genes to predict HRD status with a high AUROC using our methodology, suggesting that HRD may have a widespread effect on the expression of many genes across the transcriptome (Supp. Fig. 5A), and this was also supported by our differential expression analysis where there was a strong enrichment of low *p*-values across the transcriptome (Supp. Fig. 5B).

### Validation of the HRD200 classifier in other cohorts

**Fig. 3:**
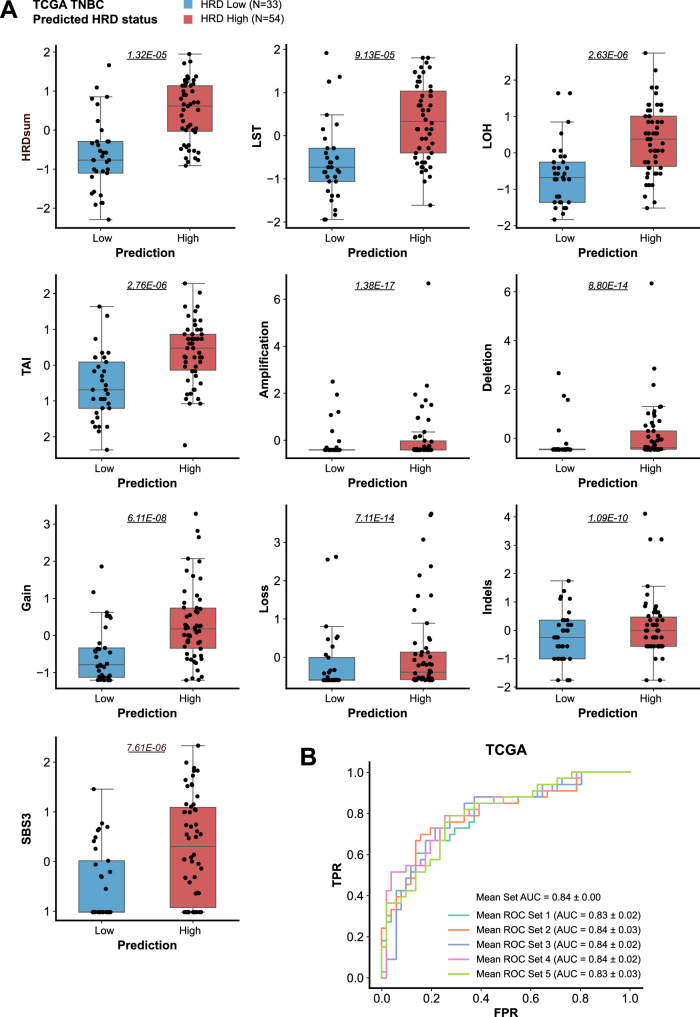
Validation of the HRD200 classifier in the TCGA cohort. **A** Comparisons of normalized scores for telomeric allelic imbalance (TAI), large-scale transitions (LST), loss-of-heterozygosity (LOH), COSMIC single base substitution mutational signature 3 (SBS3), copy number amplifications, deletions, gain, and loss, and short insertions and deletions (indels) between TCGA TNBC samples classified by the HRD200 classifier as HRD Low (*n* = 31, in blue) and samples classified as HRD High (*n* = 56, in red). All scores were scaled using z-scores, and the indels score was also log-transformed prior to scaling. Box and whiskers plots were constructed with boxes indicating 25th percentile, median (centre line) and 75th percentile, and whiskers showing the maximum and minimum values within 1.5 times the inter-quartile range from the edge of the box, with outliers shown. **B** Receiver operating characteristic (ROC) curves of false positive rate (FPR) and true positive rate (TPR) showing the performance of the HRD200 composite classifier in predicting “ground truth” HRD High status in TCGA TNBC samples that was determined by consensus hierarchical and k-means clustering of the variables included in (A). The ROC curves for each of the five component model sets are shown separately.

Next, we examined the ability of our HRD200 classifier to predict HRD-associated genomic features in other cohorts. First, we used our classifier to predict the HRD status of 87 TNBC tumours in The Cancer Genome Atlas (TCGA) dataset (TCGA 2012) for which mutational signature data was available. Comparison of the samples classified as HRD High versus HRD Low revealed that the HRD High samples had significantly higher scores for all HRD-associated variables including LOH, TAI, LST, short indels, CNAs, and mutational signature SBS3 (Fig. 3A, Supp. Fig. 6), suggesting that our classifier was able to successfully segregate samples with HRD-associated features in the TCGA TNBC cohort. We also compared the classifier predictions to a “ground truth” HRD status for the samples derived using the same methodology as applied to the MyBrCa cohort (consensus hierarchical and k-means clustering of HRD-associated variables) and found that the classifier was able to predict HRD status in TCGA TNBCs with an AUROC of 0.84 (F1 = 0.70, precision = 0.63, recall = 0.86). Using the same 0.5 probability cutoff as the MyBrCa cohort analyses above, our classifier designated 54 of the 87 samples as HRD High and 33 as HRD Low.

We also tested our classifier on 73 TNBC samples from the Nik-Zainal (2016) WGS cohort and 306 TNBC samples from the METABRIC cohort, although in both cases we had to reduce the number of genes included in the classifier and retrain the classifier as expression data was not available for some genes (see Methods). In the Nik-Zainal cohort, the samples designated as HRD High by our retrained classifier had significantly higher scores for SBS 3, rearrangement signatures 3 and 5, and HRD index scores (Supp. Fig. 7A). We also found that our retrained classifier could predict HRD classification by HRDetect at an AUROC of 0.71 (F1 = 0.57, precision = 0.72, recall = 0.67; Supp. Fig. 7B). Similarly, in the METABRIC cohort, the samples designated as HRD High by our retrained classifier had significantly higher scores for SBS 3 and copy number aberrations (Supp. Fig. 7C), and we found that our retrained classifier could predict consensus hierarchical and k-means clustering of these variables at an AUROC of 0.80 (F1 = 0.81, precision = 0.69, recall = 0.59; Supp. Fig. 7D).

### Validation of the HRD classifier using a NanoString platform

Next, we evaluated the robustness of the HRD200 classifier with regards to different methods of measuring gene expression, as well as to the use of formalin-fixed paraffin-embedded (FFPE) tissue rather than fresh frozen tissue. We did this because both RNA-seq data as well as fresh frozen tumour tissue are expensive and difficult to obtain as part of routine clinical practice.

**Fig. 4:**
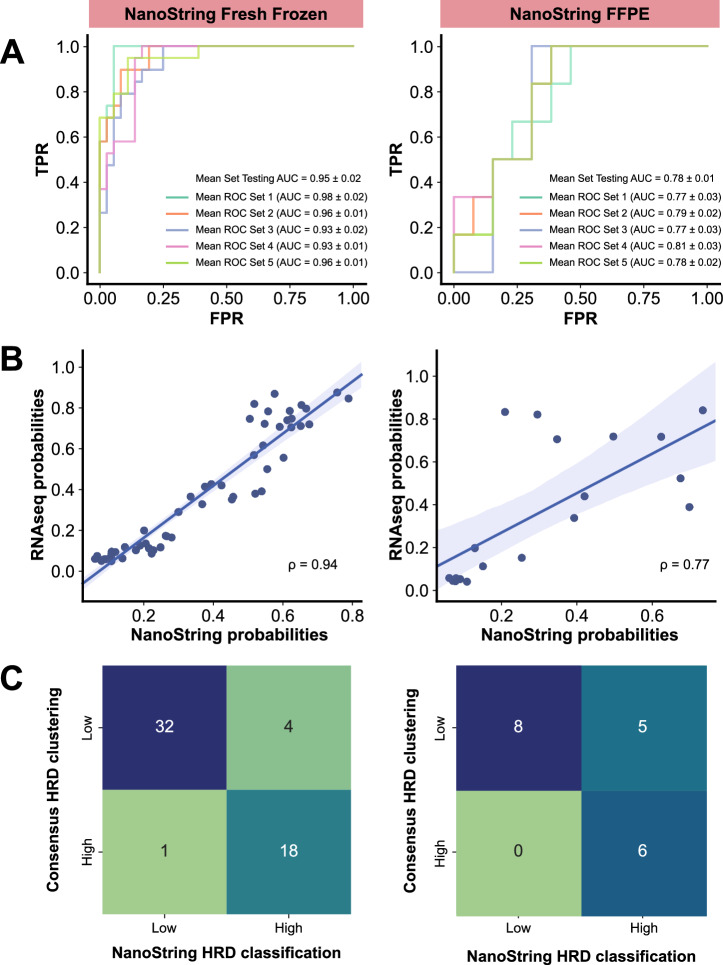
Validation of the HRD classifier on the NanoString nCounter platform. **A** Receiver operating characteristic (ROC) curves showing the performance of HRD200 classifier (retrained using a 36 gene subset) in predicting HRD High status from NanoString nCounter gene expression data from fresh frozen (left, *n* = 55) and FFPE (right, *n* = 19) samples from the MyBrCa TNBC cohort, using our consensus unsupervised clustering results as the ground truth. The ROC curves for each of the five component model sets are shown separately. **B** Comparison of the HRD High probabilities given by the HRD200 classifier for RNAseq (y-axis) and NanoString (x-axis) fresh frozen (left) or FFPE (right) matched samples. Also show are Spearman’s correlation coefficient (ρ) for each comparison. **C** Confusion matrices comparing HRD200 classification of samples using NanoString nCounter data from fresh frozen (left) or FFPE (right) samples to “ground truth” HRD status by consensus unsupervised clustering.

To evaluate the performance of the HRD200 classifier with a different gene expression measurement method, we used data from the NanoString nCounter platform^34^, which uses direct digital detection of mRNA molecules to generate gene-level transcript counts. We were able to obtain gene expression data for 36 genes of our gene set from 61 fresh frozen tissue samples as well as 23 FFPE tissue samples from the same cohort of MyBrCa TNBC patients described above, using a custom NanoString nCounter CodeSet. These data were then inputted into a version of our HRD200 classifier that was optimized for the 36 genes, and the results were compared to the HRD classification from high-throughput sequencing data. Using the consensus clustering results as the ground truth, the NanoString-based classification had an AUROC of 0.95 for fresh frozen tissue and 0.78 for FFPE (Fig. 4A) for the samples with both consensus cluster and NanoString data (n = 55 and n = 19 for fresh frozen and FFPE samples, respectively). Additionally, the probabilities for any given sample being classified as HRD High from RNAseq and NanoString data were highly correlated, with a Spearman’s correlation coefficient (ρ) of 0.94 when comparing RNASeq and fresh frozen NanoString results, and a (ρ) of 0.77 when comparing RNAseq and FFPE NanoString results (Fig. 4B). Pairwise comparisons of the NanoString and consensus clustering results revealed an overall accuracy of 0.91 and an F1 score of 0.88 for fresh frozen tissue, and an accuracy of 0.74 and an F1 score of 0.71 for FFPE tissue (Fig. 4C, Supp. Fig. 8), when using probability cutoffs that maximized F1 scores. Overall, this suggests that our HRD200 classifier was robust even when used with expression data from a different platform, and also when used with smaller subsets of genes compared to the original gene set.

## Discussion

Here, we describe the development and validation of a gene expression classifier for homologous recombination deficiency in Asian TNBC. Using mutation load and other genomic features, we were able to sort our TNBC samples into two clusters - HRD high and HRD low, and we derived a gene set associated with HRD status using gene expression analyses. Using that gene set, we subsequently developed an ensemble machine learning model classifier (HRD200) that could discriminate between samples with high versus low HRD with good accuracy and AUROC from gene expression data. The classifier also had the ability to segregate samples according to genomic features associated with HRD in TNBC validation cohorts from TCGA, METABRIC, and Nik-Zainal (2016). Importantly, we also found a very high concordance rate in the classification results when using an alternative measure of gene expression (NanoString), as well as when using FFPE material instead of fresh frozen tissue, even with small subsets of the gene panel, suggesting that the HRD200 classifier could be robust for real-world clinical use situations.

In the TNBC setting, we found a large cluster of samples with high HRD-associated genomic features and mutational signatures. This suggests that there may be a significant number of Asian TNBC patients who may benefit from therapies that target the HRD pathway, such as platinum chemotherapy or PARP inhibitors. Thus, tools to detect HRD in tumour samples may have clinical utility as a non-gBRCAm biomarker to select for Asian breast cancer patients with high HRD who may benefit from treatment with platinum chemotherapy or PARP inhibitors.

The training and validation of the HRD classification tool described in this paper relies in part on measures of the mutational signature SBS3 in each tumour sample. The COSMIC mutational signature SBS3 is a well-studied cancer mutational signature that has been validated in orthogonal techniques^35,36^, and its HRD-driven etiology has been experimentally confirmed^37^. The use of HRD-associated mutational signatures to predict “BRCAness” and thus response to PARP inhibitors and platinum chemotherapy has received significant attention in recent years, with tools such as HRDetect^19^ and CHORD^38^ demonstrating that HRD-associated mutational signatures can be used to predict BRCA1/BRCA2-deficiency in breast tumours as well as survival of breast cancer patients^39^. The clinical utility of such tools in a breast cancer setting is being tested in ongoing clinical trials; however, one significant drawback is that these methods usually require whole-genome sequencing of tumour samples, which may be prohibitively expensive and time-consuming, particularly in low-resource settings. By using gene expression signatures, our method for predicting HRD in tumour samples may offer a useful alternative.

As mentioned above, other gene expression signatures related to HRD have also been previously described—Peng and colleagues (2014)^30^ described a set of 230 genes associated with homologous recombination DNA repair in a microarray analysis of a nonmalignant human mammary epithelial cell line that predicts clinical outcome in cancer patients, while a different 77-gene panel of “*BRCA1*ness” (derived from microarray and MLPA analyses of TNBC samples) was significantly associated with response to PARP inhibitor treatment in the I-SPY 2 breast cancer clinical trial^31,32^. Interestingly, there appears to be little overlap between the different gene panels—only one out of the 217 genes in our gene set are included in Peng’s 230-gene set, and only 15 of the 217 genes in our study are included in the 77-gene “BRCA1ness” panel, with zero genes common across all three sets. The lack of overlap may reflect the large differences in methodology or study population. Nonetheless, when the same machine learning approach was used, all three gene sets were almost equally predictive for HRD status in our dataset, with very high predictive value across the board. This suggests that the machine learning methodology used may be more important than the specific gene set for researchers to be able to derive accurate predictions of HRD status from gene expression data, as long as the gene set used retains some association with HRD in the study population.

Taken together, we believe that the HRD200 classifier, implemented as a NanoString-based test, may have clinical utility as a non-*BRCA*m biomarker to select for patients with high HRD who may benefit from treatment with PARP inhibitors. Further development of the classifier is required to determine if HRD200 can correctly identify patients sensitive to PARP inhibitor therapy in real-world clinical settings.

## Methods

### Data description

Genomic sequencing data for this project was taken primarily from 94 TNBC samples included in the MyBrCa cohort tumour sequencing project. In brief, this included whole-exome sequencing (WES) and RNA-sequencing (RNA-seq) data collected from biobanked breast tumours of female patients from two hospitals – Subang Jaya Medical Centre in Subang Jaya, Malaysia, and Universiti Malaya Medical Centre in Kuala Lumpur, Malaysia, and analysed together with available clinical data. The cohort data and sequencing methods are described in full in Pan et al.^24^ and associated papers^24,25,40^. We also included an additional 35 TNBC samples that were not part of the original cohort description, for a total sample size of 129 MyBrCa TNBC samples. These samples were obtained and processed in largely the same way as the previous MyBrCa samples, with the only difference being the use of the Illumina NovoSeq 6000 as the sequencing platform instead of the Illumina HiSeq 4000. The sequencing coverage and quality statistics of WES and RNA-seq data for each new sample are summarized in Supp. Tables 1A and 1B, respectively. Additional validation data from TCGA and METABRIC TNBC samples were downloaded from the NIH Genomics Data Portal and the European Genome-phenome Archive, respectively.

Patient recruitment and sample collection was reviewed and approved by the Independent Ethics Committee, Ramsay Sime Darby Health Care (reference no: 201109.4 and 201208.1), as well as the Medical Ethics Committee of the University Malaya Medical Centre (reference no: 842.9). Written informed consent to participation in research was given by each individual patient.

### Transcriptomic data processing

Raw RNA-Seq reads were mapped to the hs37d5 reference human genome, and gene-level read counts were quantified using featureCounts (v. 1.2.31) with the Homo sapiens GRCh37.87 human transcriptome genome annotation model.

### Mutational analyses

To call SNVs, we used positions called by Mutect2 with following filters: minimum 10 reads in tumour and 5 reads in normal samples, OxoG metric less than 0.8, variant allele frequency (VAF) 0.075 or more, *p*-value for Fisher’s exact test on the strandedness of the reads 0.05 or more, and S_AF_ more than 0.75. For positions that are present in 5 samples or more, we removed two positions that were not in COSMIC and in single tandem repeats. We also removed variants that have VAF at least 0.01 in gnomAD, and considered only variants that are supported by at least 4 alternate reads, with at least 2 reads per strand. For indels, we also required the positions to be called by Strelka2. Variants were annotated using Oncotator version 1.9.9.0.

### Determination of HRD status

Genomic features from WES and sWGS data were used in a clustering step to group the TNBC samples into 2 groups: HRD high and HRD low. The genomic features used include telomeric allelic imbalance (TAI), loss of heterozygosity (LOH), large-scale transitions (LST), copy number amplification, copy number gain, copy number loss, copy number deletion, indel counts, and COSMIC mutational signature SBS3 scores. TAI, LOH and LST scores were determined using the scarHRD R package (v. 0.1.1)^41^ on allele-specific copy number profiles derived by Sequenza (v. 2.2) from paired tumour-matched normal WES bam files. The prevalence of the HRD-associated single base-pair substitution (SBS) mutational signature 3 from COSMIC (SBS3) was determined using deconstructSigs (v.1.8.0), restricted to samples with at least 15 SNVs. Scores for copy number amplification, gain, loss, and deletion were obtained using the QDNASeq R package (v. 1.22) on shallow-whole genome sequencing bam files. Scores for each feature were normalized using z-scores before clustering, except for indel counts which were log-transformed, then all the scores were rescaled. K-means clustering and hierarchical clustering were performed using the Python packages “scikit-learn” (v. 1.2.1) and “scipy” (v. 1.12.0) respectively. Only samples that reached consensus between the two clustering algorithms were selected for further analysis, and the consensus clustering results were assigned as the HRD status of each sample.

### Differential expression analyses

Gene-level count matrices were normalised using the “Trimmed Mean of M-values” method implemented in the edgeR (v. 3.20.9) R package. The normalized count matrices were then transformed into log_2_ counts-per-million (CPM) values using the “cpm” function from the edgeR package in R. The count matrix was first filtered to remove very lowly- and non-expressed genes. Differentially expressed genes were determined by empirical Bayes moderation of the standard errors towards a common value from a linear model fit of the transformed count matrices as implemented in the limma package, with the threshold for differential expression set as false discovery rate (FDR) < 0.001 and absolute log fold change > 0.2.

### Pathway analysis

Over-representation analysis using KEGG and Reactome pathway-based sets as well as gene-ontology (GO) based sets was conducted using ConsensusPathDB (http://cpdb.molgen.mpg.de, accessed 21 April 2022) using the human database and ENSEMBL identifiers. For GO-based sets, the search was restricted to gene ontology level 2 and level 3 categories only.

Pathway analysis was conducted using gene set enrichment analysis (GSEA), as implemented in the Broad Institute GSEA Java executable (v 4.2.3), using the MSigDB Hallmark gene sets, as well as the KEGG gene sets, as implemented in the GSEA program using default options.

### Determination of germline *BRCA* mutation status

Carriers of deleterious pathogenic germline variants in *BRCA1* and *BRCA2* in the MyBrCa cohort were identified from targeted sequencing conducted as part of the BRIDGES study^42^. LOH and biallelic status of the germline variants were taken from Ng et al. ^25^. Each carrier was independently confirmed with Sanger sequencing.

### Classifier architecture

The machine learning framework was implemented in Python (v. 3.9.6) using the libraries “scikit-learn”, “scipy”, “numpy” (v. 1.26.4), “pandas” (v. 1.5.3). The input dataset for the classifier consisted of RNA-seq gene expression data quantified as TMM and log_2_ normalized counts per million (CPM), along with the HRD classification of each sample.

Our classifier architecture consisted of a double loop system (Supp. Fig. 1). In the outer loop, the input data was split into training and testing sets following a 70/30 ratio using a one-fold stratified shuffle split repeated five times with different seeds, resulting in five sets of training and testing data that were passed into the inner loop. The inner loop combined two classifier pipelines for Support Vector Machine and Random Forest algorithms, respectively, with the probability that a sample is HRD High being the average score of both pipelines. The inner loop pipeline architecture was adapted from Sammut et al. (2021)^33^ and has a feature selection step built into the classifier pipeline prior to the classification model, consisting of z-score scaling, k-best selection and collinearity removal. Within the inner loop, the hyperparameters were optimized using a five-fold randomized cross-validation (CV) search that maximizes the area under the receiver operating characteristic (AUROC). This randomized CV search tested 1000 random combinations sampled from the specified hyperparameter distributions. The optimization was repeated five times as part of the cross-validation step, and the final scores for the inner loop were the average scores of the five-fold CV. After training, the models were validated against their testing datasets to determine the AUROC for each set of data in the outer loop. Lastly, the AUROC scores from each repetition were averaged to get the final reported AUROC for the entire ensemble classifier. The final ensemble classifier is essentially composed of five sets of five SVM and RF models (25 models in total for each algorithm), and the scores generated by the ensemble classifier are the average scores across all five sets. The optimized hyperparameters and selected features for each model are reported in the supplementary material. This final ensemble classifier was used for further validation, referred to below as the “MyBrCa model”.

### Validation on other cohorts

The classifier was validated using gene expression data from TNBC samples from other cohorts, including TCGA, the Molecular Taxonomy of Breast Cancer International Consortium (METABRIC) cohort^27,28^, and the Nik-Zainal (2016)^29^ (NZ-560) cohort from the International Cancer Genome Consortium (ICGC). Because the individual cohort datasets did not always contain all the genes used in the model training, the models used in each validation were retrained on the MyBrCa data using the available genes for that cohort. The TCGA cohort RNA-seq data was downloaded from the GDC Data Portal and included all 217 genes used in the MyBrCa model. The METABRIC cohort, unlike our other cohorts, includes microarray data rather than RNA-seq data, and includes only 146 of the genes used in the MyBrCa model. Gene expression data for the METABRIC cohort was downloaded from the European Genome-phenome Archive. For the NZ-560 cohort, we used the log2 FPKM gene expression values from RNA-seq data that was reported in the original publication, but data was available for only 164 of the genes used in MyBrCa model. Gene expression values from each cohort were normalized using z-score scaling and quantile normalization separately for each cohort before classification. F1 score, precision, and recall values were calculated using the HRD200 probability threshold that maximized F1 score.

### RNA extraction

RNA from tumour samples was extracted using the QIAGEN miRNeasy Mini Kit with a QIAcube, according to standard protocol. Total RNA was quantitated using a Nanodrop 2000 Spectrophotometer and RNA integrity was measured using an Agilent 2100 Bioanalyzer.

### NanoString validation

For the NanoString validation, we used data from a custom CodeSet developed for the NanoString nCounter platform. This custom CodeSet included 35 genes from our gene set and 3 housekeeping genes used for data normalization. We obtained NanoString nCounter read counts for these genes from 61 fresh frozen samples and 23 FFPE samples from the MyBrCa TNBC cohort. Expression for this gene set was measured on an nCounter MAX Analysis System, and the raw data was processed and normalized using the NanoString’s proprietary nSolver (v. 4.0) software before being exported as a normalized gene expression matrix text file for processing by the machine learning classifier, which was retrained using only the 35 genes included in the NanoString data. The NanoString gene expression values were normalized using z-score scaling and quantile normalization before classification. The fresh frozen and FFPE samples were normalized separately.

### Statistical analyses

All box and whiskers plots in the figures are constructed with boxes indicating 25th percentile, median and 75th percentile, and whiskers showing the maximum and minimum values within 1.5 times the inter-quartile range from the edge of the box, with outliers shown.

### Supplementary information

Supplemental Figures 1-8

Supplemental Table 1

Supplemental Table 2

Supplemental Table 3

Supplemental Table 4

Supplemental Table 5

Supplemental Table 6

Supplemental Table 7

## Data Availability

The WES and RNA-seq data generated in this study are available in the European Genome-phenome Archive under accession number EGAS00001006518. Previously published data from Pan et al. ^24^ are available in EGA under accession numbers EGAS00001004518. Access to controlled patient data will require the approval of the Data Access Committee. Further information is available from the corresponding author upon request.

## Code availability

The code used in the machine learning model training and validation is available on GitHub at https://github.com/ziching18/HRD200.
