# Supplementary material for: Gene expression signature for predicting homologous recombination deficiency in triple-negative breast cancer
Source: NPJ Breast Cancer. 2024 Jul 19;10:60. doi: 10.1038/s41523-024-00671-1 (PMC11271517; doi:10.1038/s41523-024-00671-1)
Supplement: Supplementary file 1 — Supplemental Figures 1-8 [file 41523_2024_671_MOESM1_ESM.pdf]

Gene expression signature for predicting homologous recombination deficiency in triple-negative breast cancer

Jia-Wern Pan, Zi-Ching Tan, Pei-Sze Ng, Muhammad Mamduh Ahmad Zabidi, Putri Nur Fatin, Jie-Ying Teo, Siti Norhidayu Hasan, Tania Islam, Li-Ying Teoh, Suniza Jamaris, Mee-Hoong See, Cheng-Har Yip, Pathmanathan Rajadurai, Lai-Meng Looi, Nur Aishah Mohd Taib, Oscar M. Rueda, Carlos Caldas, Suet-Feung Chin, Joanna Lim, Soo-Hwang Teo

Supplemental Material

Contents

I. Supplemental Figures 1 to 8
II. Table Legends for Supplemental Tables 1 to 7

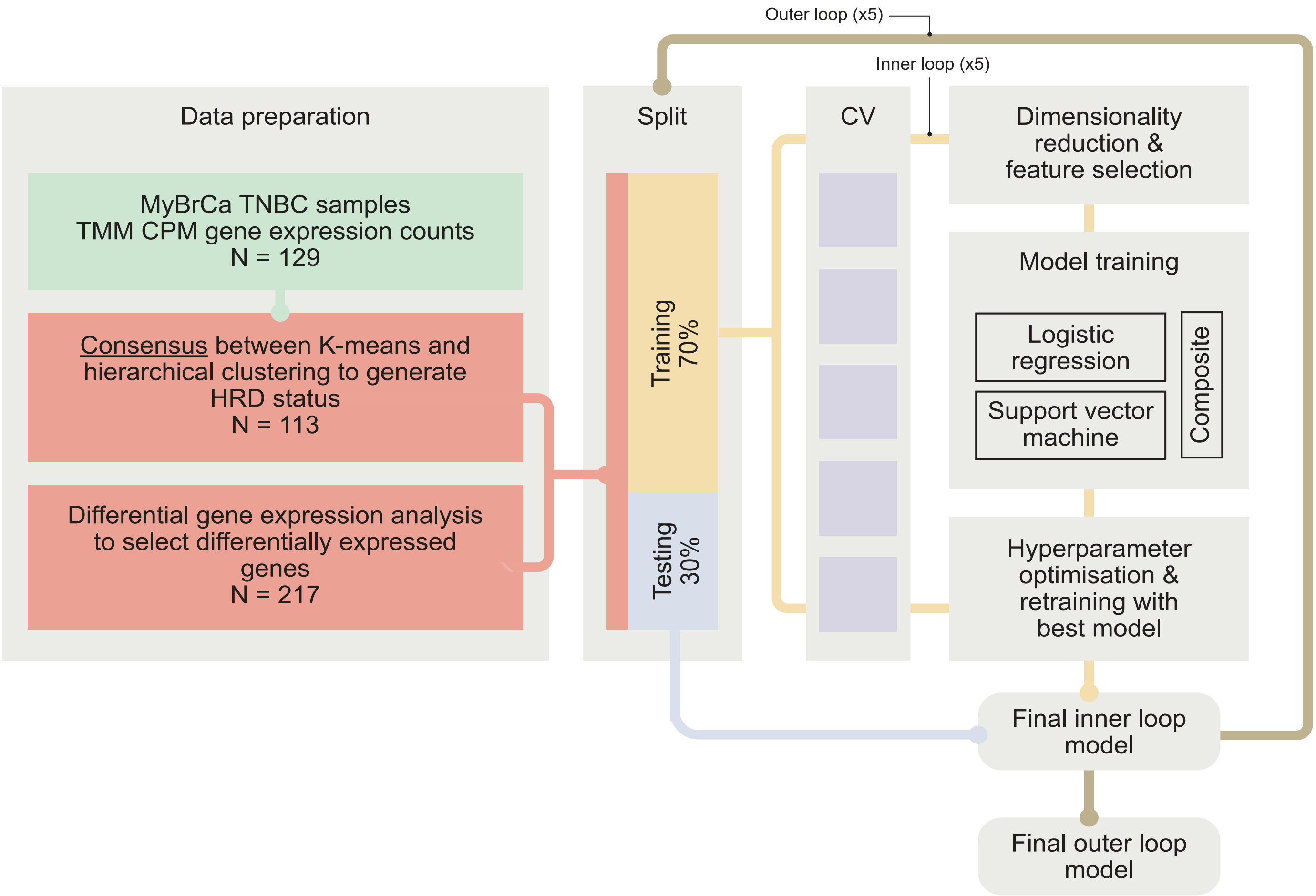

Supp. Fig. 1. Diagram showing the architecture of the ensemble classifier.

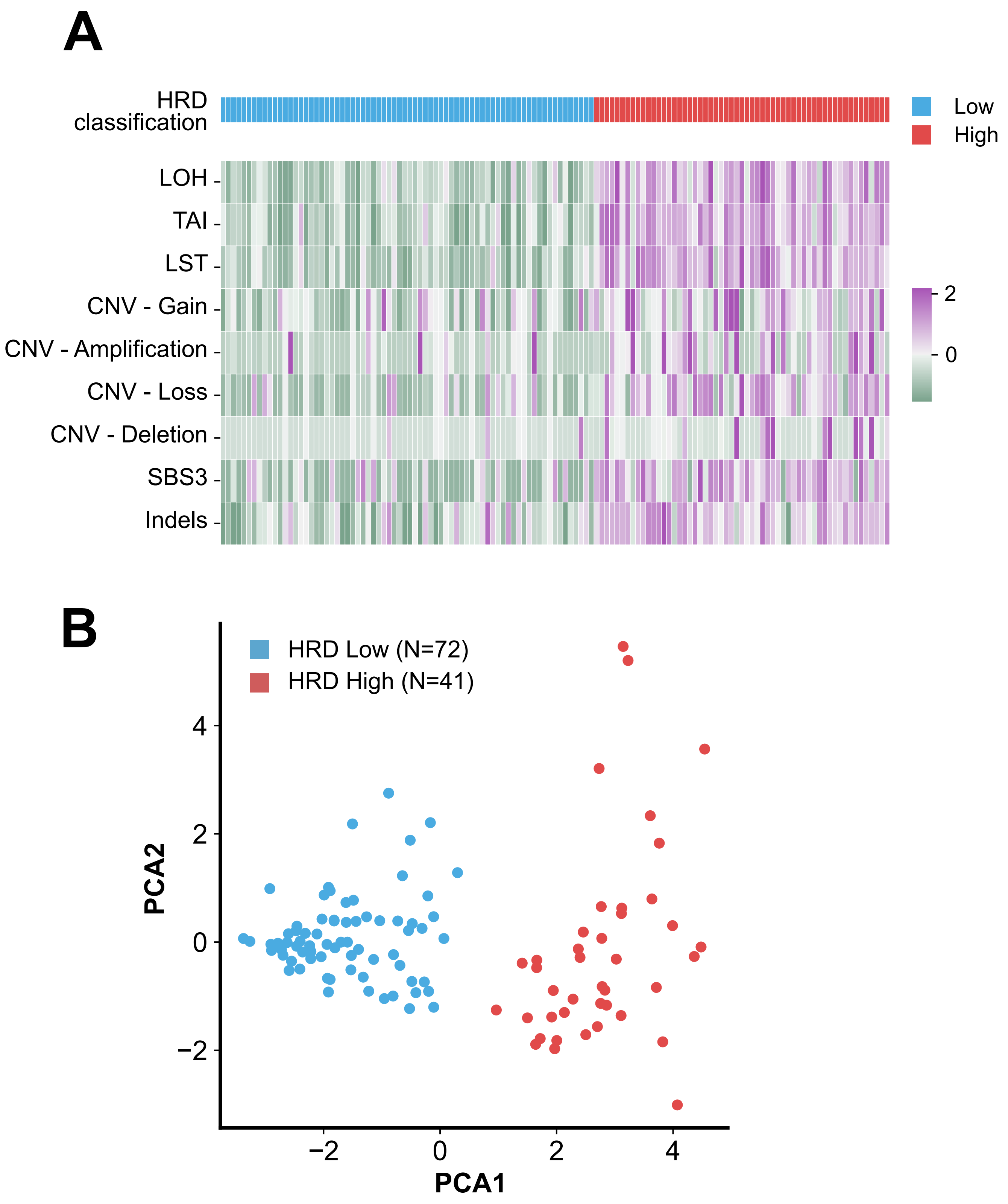

Supp. Fig. 2. K-means clustering of HRD-associated variables in the MyBrCa cohort. A. HRD classification of MyBrCa samples by k-means clustering (k=2) of MyBrCa samples. Heatmap shows the normalized scores of each sample for the HRD-associated variables used in the clustering. **B.** Principal component analysis of the MyBrCa cohort samples using the HRD-associated variables used in k-means clustering. The color of each dot indicates the HRD classification of the associated sample by k-means clustering.

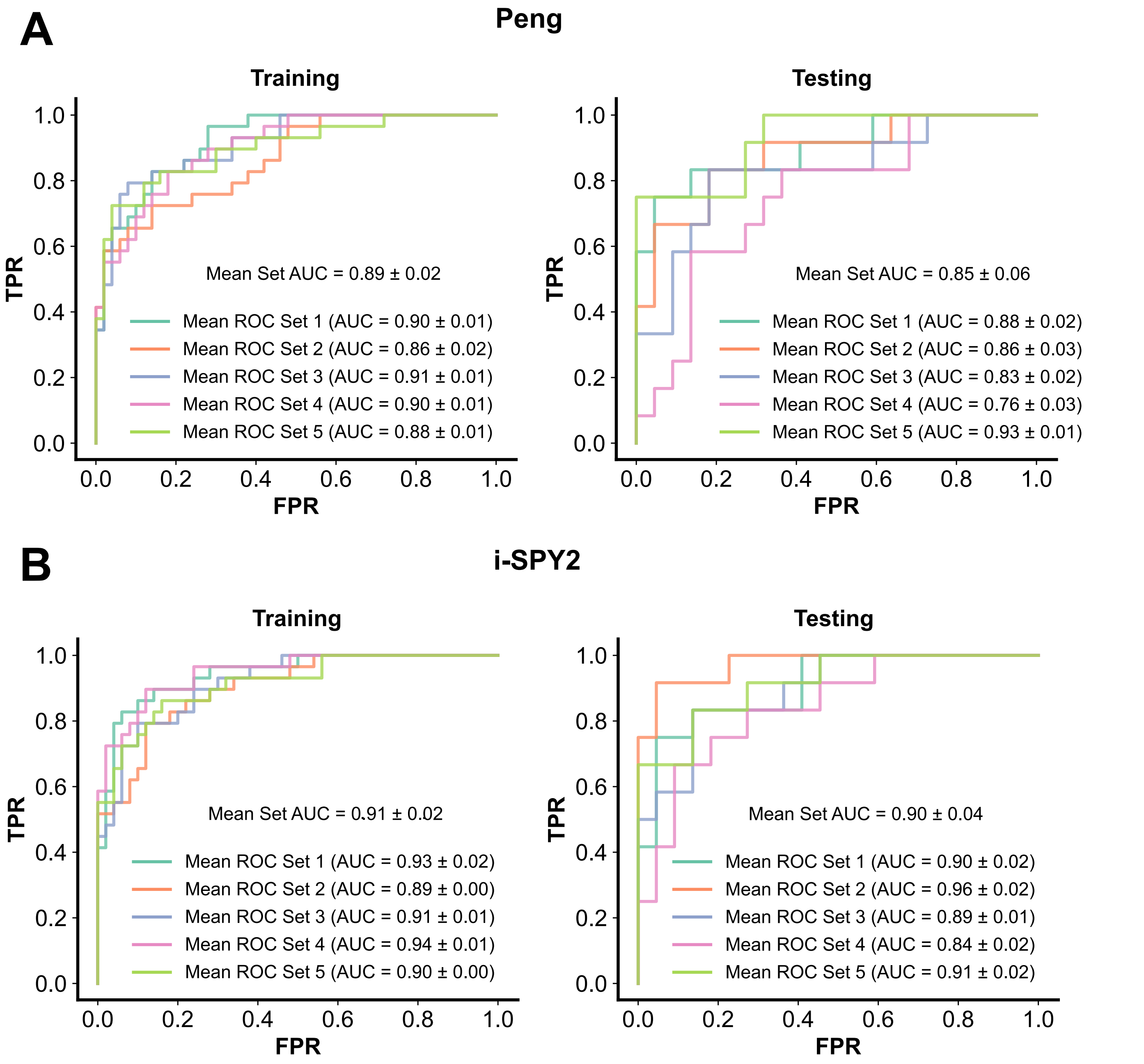

Supp. Fig. 3. Area under receiver operating characteristics curve (AUROC) for training and testing datasets of our composite classifier trained using **(A)** the set of 230 genes associated with HRD from Peng et al. (2014), and **(B)** the set of 77 genes associated with BRCA1ness in the i-SPY2 cohort (Severson et al., 2017).

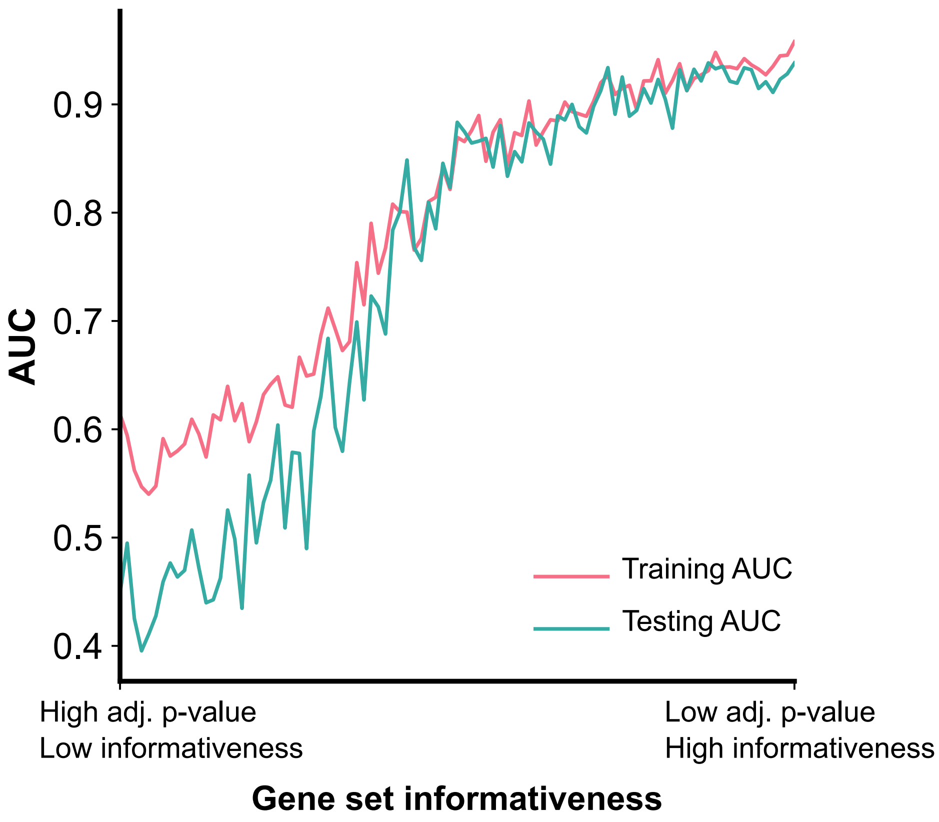

Supp. Fig. 4. The figure shows the area under the ROC curve (AUC) of 95 ensemble classifiers trained with gene sets of increasing informativeness using a sliding window approach, from gene sets (consisting of 200 genes each) that had high mean adjusted p-values (in our differential expression analysis comparing samples classified as High versus Low HRD) on the left, to gene sets that had low mean adjusted p-values on the right. Both training and testing AUCs from the composite models are shown.

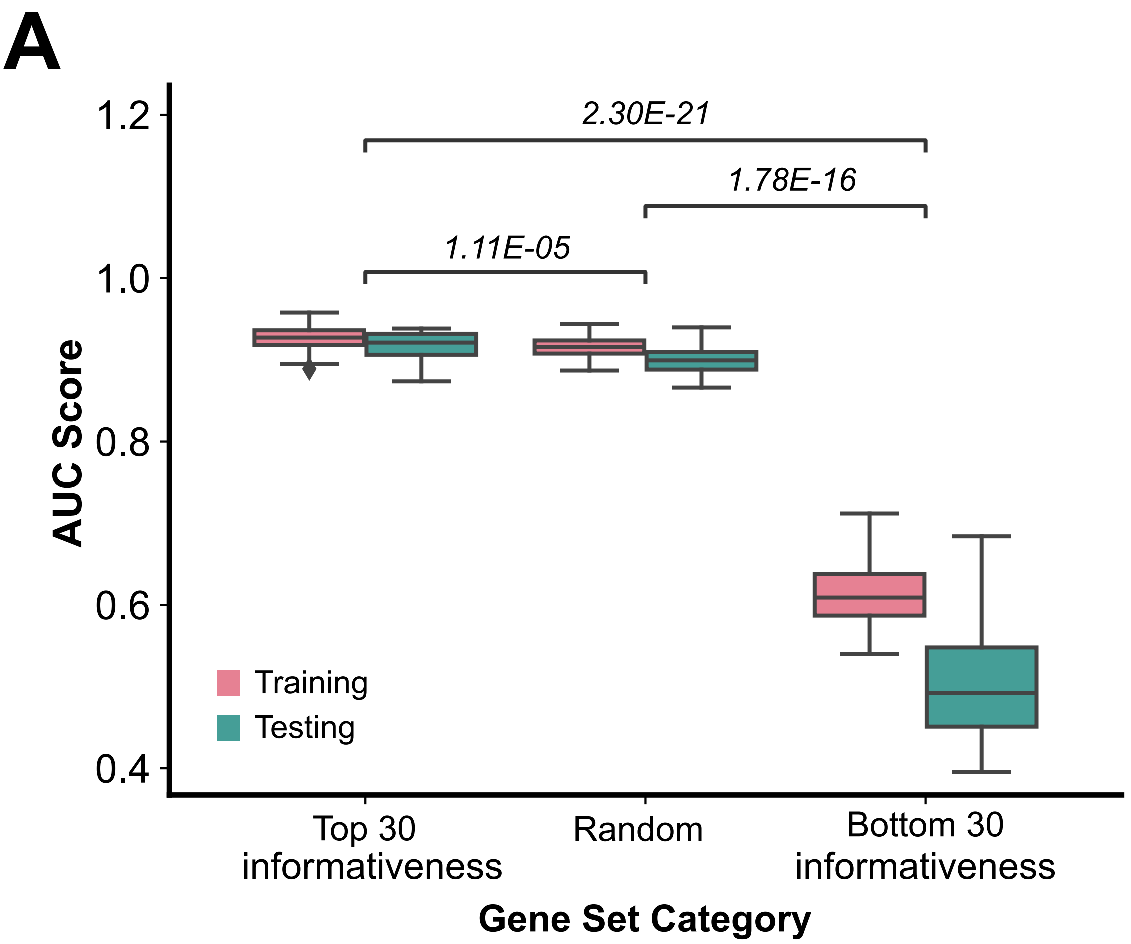

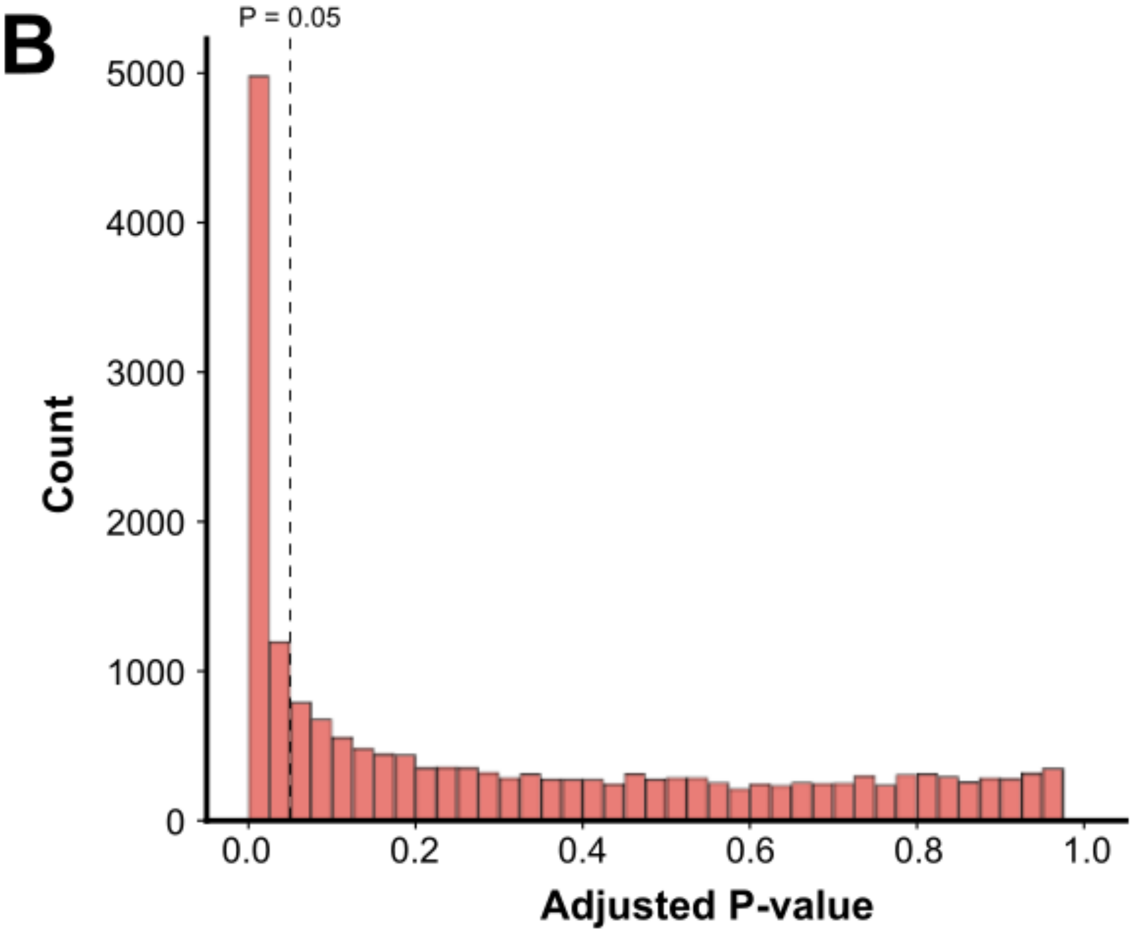

Supp. Fig. 5. Gene set validation. A. Distribution of AUC scores for training and testing datasets of ensemble models trained with different sets of 200 genes, comparing the top thirty gene sets by informativeness of the genes according to differential gene expression analyses (left), random gene sets (middle), and the bottom thirty gene sets (right). P-values shown are for Mann Whittney U tests comparing the testing datasets of the three categories. **B.** Distribution of Benjamini-Hochberg adjusted P-values across all expressed genes for a differential expression analysis comparing HRD High to HRD Low samples.

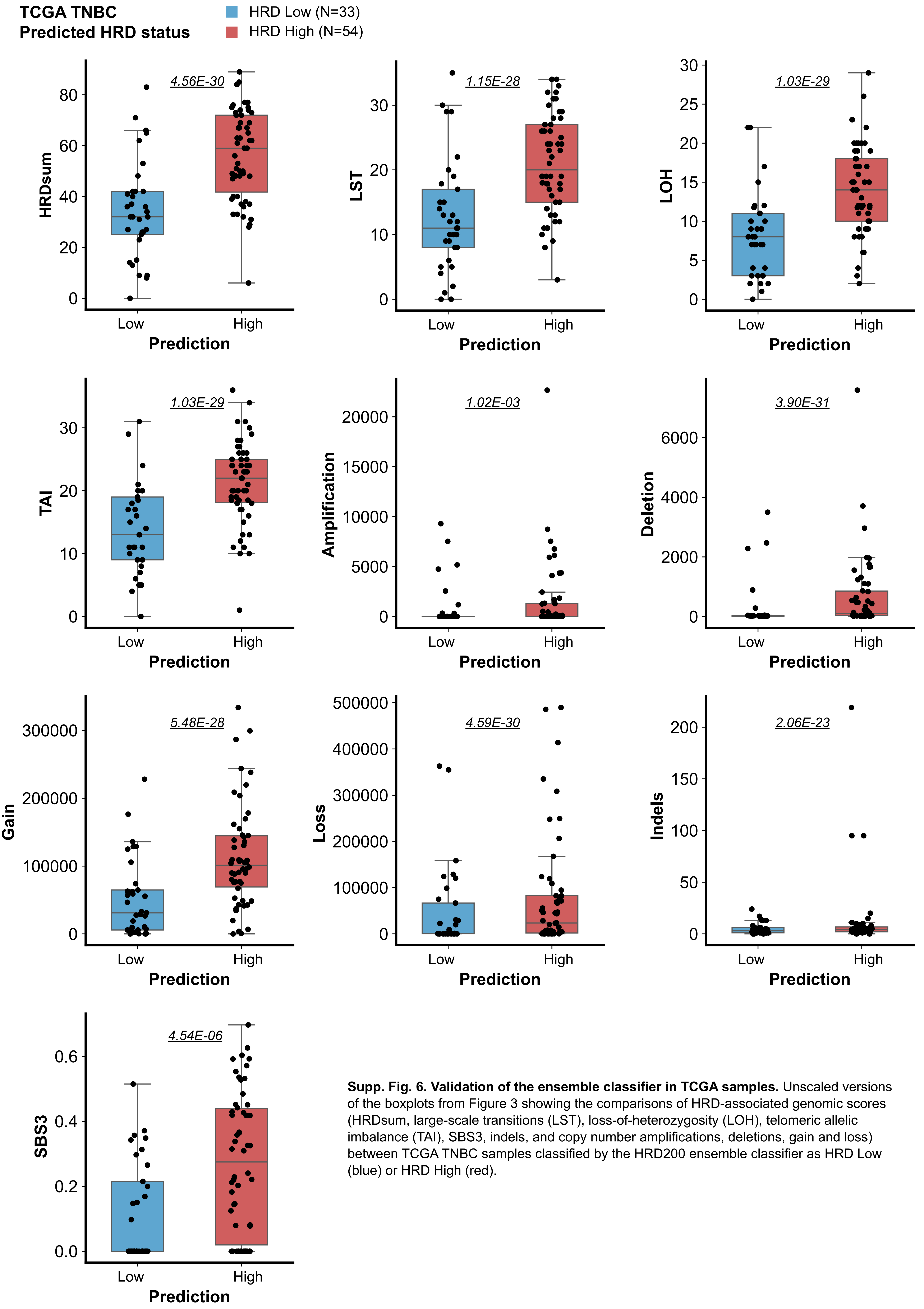

Supp. Fig. 6. Validation of the ensemble classifier in TCGA samples. Unscaled versions of the boxplots from Figure 3 showing the comparisons of HRD-associated genomic scores (HRDsum, large-scale transitions (LST), loss-of-heterozygosity (LOH), telomeric allelic imbalance (TAI), SBS3, indels, and copy number amplifications, deletions, gain and loss) between TCGA TNBC samples classified by the HRD200 ensemble classifier as HRD Low (blue) or HRD High (red).

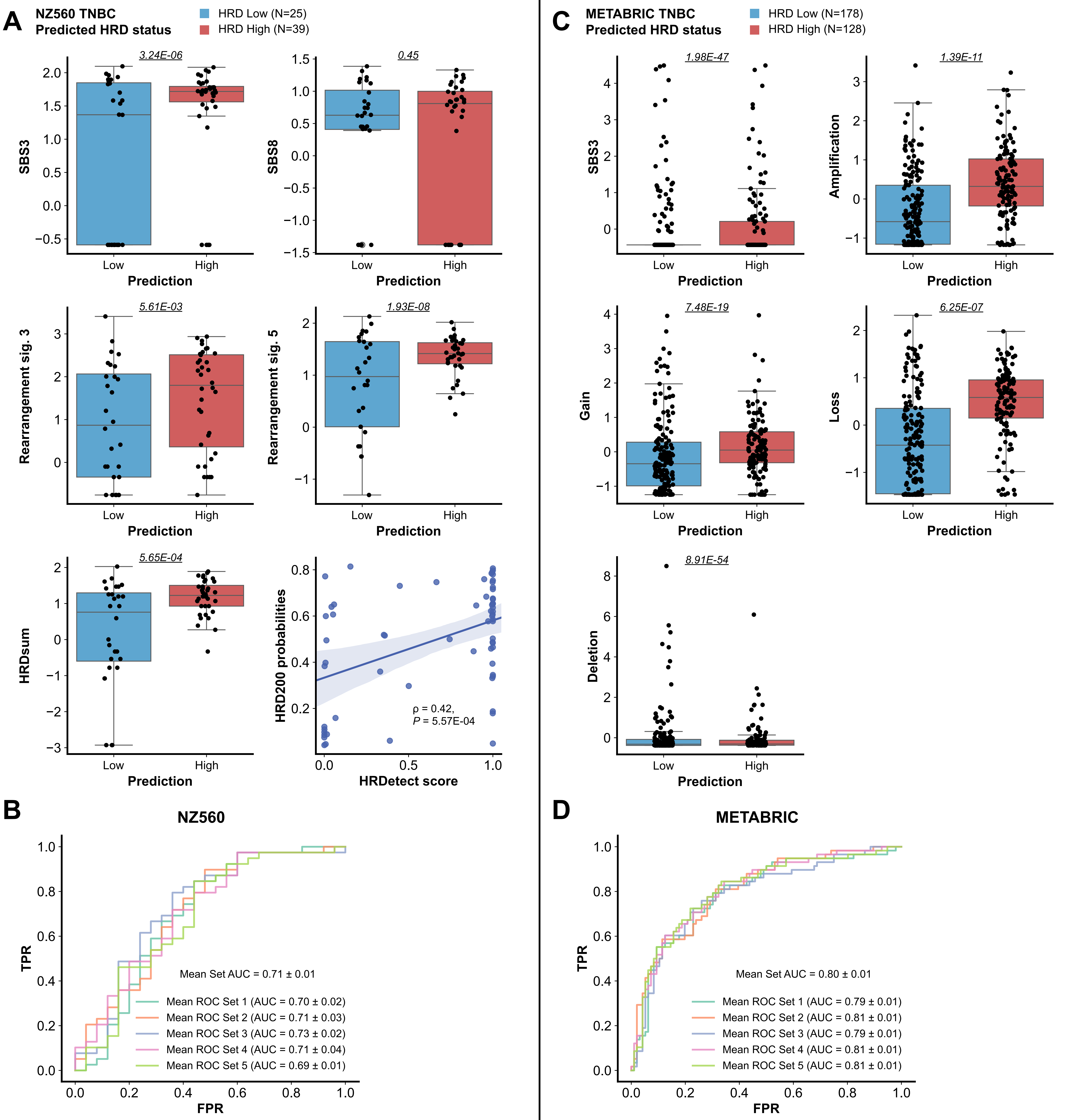

A
NZ560 TNBC
Predicted HRD status
HRD Low (N=25)
HRD High (N=39)
3.24E-06
0.45
SBS3
SBS8
Prediction
5.61E-03
1.93E-08
Rearrangement sig. 3
Rearrangement sig. 5
5.65E-04
HRDsum
HRD200 probabilities
HRDetect score
ρ = 0.42,
P = 5.57E-04
C
METABRIC TNBC
Predicted HRD status
HRD Low (N=178)
HRD High (N=128)
1.98E-47
1.39E-11
Amplification
7.48E-19
6.25E-07
Gain
Loss
8.91E-54
Deletion
B
NZ560
Mean Set AUC = 0.71 ± 0.01
Mean ROC Set 1 (AUC = 0.70 ± 0.02)
Mean ROC Set 2 (AUC = 0.71 ± 0.03)
Mean ROC Set 3 (AUC = 0.73 ± 0.02)
Mean ROC Set 4 (AUC = 0.71 ± 0.04)
Mean ROC Set 5 (AUC = 0.69 ± 0.01)
TPR
FPR
D
METABRIC
Mean Set AUC = 0.80 ± 0.01
Mean ROC Set 1 (AUC = 0.79 ± 0.01)
Mean ROC Set 2 (AUC = 0.81 ± 0.01)
Mean ROC Set 3 (AUC = 0.79 ± 0.01)
Mean ROC Set 4 (AUC = 0.81 ± 0.01)
Mean ROC Set 5 (AUC = 0.81 ± 0.01)

Supp. Fig. 7. Validation of classifier in NZ560 and METABRIC cohorts. (A) Difference in normalized scores for HRD-associated variables (SBS3 and SBS8 mutational signatures, Rearrangment signatures 3 and 5, HRD index scores) between TNBC samples from the Nik Zainal et al. (2016) WGS cohort (NZ560) that were predicted as HRD Low (blue) or HRD High (red) by our ensemble classifier. Also shown is a direct comparison between scores generated by our classifier (HRD200) and HRDetect. **(B)** AUROC of our ensemble classifier scores for NZ560 samples compared to HRD classification by HRDetect. **(C)** Difference in normalized scores for HRD-associated variables (SBS3, copy number amplifications, deletions, gains, and loss) between TNBC samples from the METABRIC cohort that were predicted as HRD Low (blue) or HRD High (red) by our ensemble classifier. **(D)** AUROC of our ensemble classifier scores for METABRIC samples compared to HRD classification by consensus k-means and hierarchical clustering of the HRD-associated variables in (C).

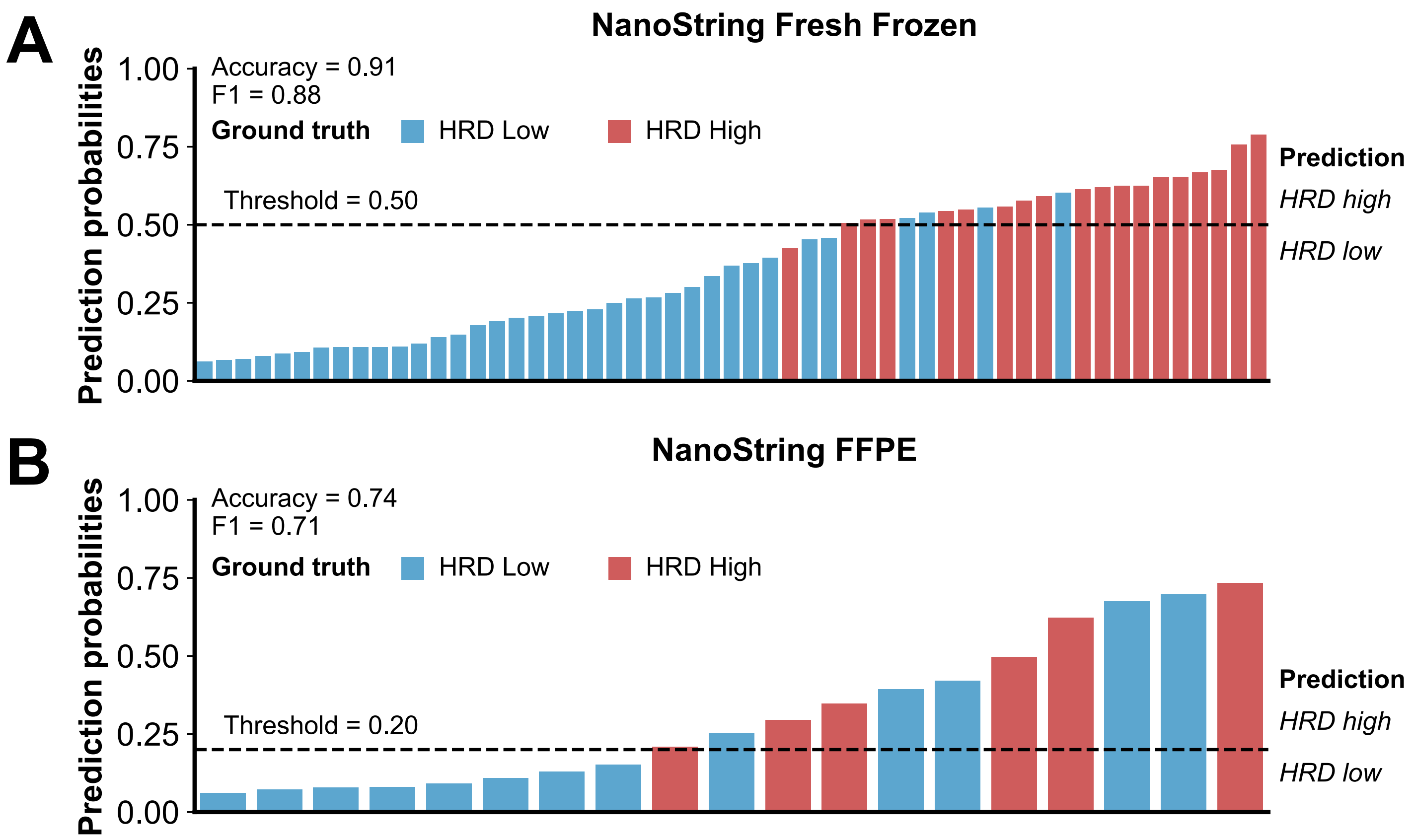

Supp. Fig. 8. HRD classification using NanoString nCounter data. Bar charts showing the probability of a sample being HRD High according to the HRD200 ensemble classifier using NanoString nCounter data from (A) fresh frozen and (B) FFPE samples from the MyBrCa TNBC cohort, compared to their HRD classification from consensus clustering of HRD-associated genomics variables (color of the bar). The probability threshold used for HRD classification of the NanoString data is indicated with a dotted line.

Table Legends for Supplemental Tables 1 to 7

Supplemental Table 1. WES and RNAseq sequencing coverage and mapping statistics for 35 MyBrCa TNBC validation samples to hs37d5.

Supplemental Table 2. Gene set to determine HRD in breast tumours. Gene start and end locations are based on the GrCh38 human reference genome.

Supplemental Table 3. Over-representation analysis of KEGG and Reactome pathways for differentially expressed genes between TNBC tumour samples from the MyBrCa training cohort with high HRD scores versus low HRD scores.

Supplemental Table 4. Over-representation analysis of gene ontology terms for differentially expressed genes between TNBC tumour samples from the MyBrCa training cohort with high HRD scores versus low HRD scores.

Supplemental Table 5. Gene set enrichment analysis of MSigDB Hallmark pathways comparing TNBC tumour samples from the MyBrCa training cohort with high HRD scores to low HRD scores.

Supplemental Table 6. Gene set enrichment analysis of KEGG pathways comparing TNBC tumour samples from the MyBrCa training cohort with high HRD scores to low HRD scores.

Supplemental Table 7. Optimized hyperparameters for SVM and Random Forest models
